# Phylogenetic analysis of *Anguilla marmorata* population in Thua Thien Hue, Vietnam based on the cytochrome C oxidase I (COI) gene fragments

**DOI:** 10.1186/s13568-020-01059-7

**Published:** 2020-07-07

**Authors:** Kieu Thi Huyen, Nguyen Quang Linh

**Affiliations:** 1 Faculty of Fisheries, University of Agriculture and Forestry, Hue University, 102 Phung Hung, Hue, 49000 Vietnam; 2 Department of Nutritional Diseases and Systems for Livestock and Aquaculture, Institute of Biotechnology, Hue University, Hue City, 49000 Vietnam

**Keywords:** *Anguilla marmorata*, Nucleotides, Animo acids, Phylogenetic tree

## Abstract

The giant mottled eel is a species with high commercial value so overfishing, river management, and water pollution have negatively affected its movement and population numbers. *Anguilla marmorata* (eel) was listed in the Vietnam Red Data Book 2007 with a description of Vulnerability. This study used a barcode technique to analyze molecular characteristics and build genetic plants based on the cytochrome c oxidase I gene segment isolated from the mitochondrial genome of 48 individuals of *A. marmorata* collected in five different ecological regions of Thua Thien Hue, Vietnam. The isolated the cytochrome c oxidase I sequence has a length of 843 nucleotides, four base nucleotides of 30.03% Thymine, 25.15% Cytosine, 27.49% Adenine, and 17.43% Guanine. The percentage of Guanine + Cytosine content (42.58%) is acceptable, lower than the Adenine + Thymine content. The replacement capacity of Adenine and Guanine is 22.45% highest, the ratio between Thymine and Guanine; Cytosine and Guanine are the lowest at 2.72%. The establishment of genetically modified plants has shown the high genetic similarity of individuals in eel populations in Thua Thien Hue. The population of *Anguilla marmorata* eels in Thua Thien Hue, Vietnam is divided into two separate groups that are guided by the migration process and specific ecological. This is particularly important in building strategies to conserve and develop the gene for eel in Vietnam and Thua Thien Hue.

## Introduction

Freshwater eels consist of 16 species, included *Anguilla marmorata*, three of which are further divided into two subspecies (Watanabe 2003; Watanabe et al. 2004, 2005). The distribution of freshwater eels are normally in temperate, tropical, and subtropical areas; which is considered to be prevalent nearly worldwide, except for the land masse adjacent to the South Atlantic and the eastern Pacific Oceans. They all have a catadromous life-history strategy, spawning in remote tropical seas with larvae that are transported back by currents to their nursery grounds in freshwater or coastal areas (Arai 2016) with distances from several hundred to thousands of kilometers (Arai 2014). During migration between oceans and freshwater during special stages of the life cycle, strong environmental changes have shaped their physiological characteristics, for example, visual sensitization, ability to smell cupping, and salinity tolerance (Williamson and Boëtius 1993). Changes in habitat conditions and distribution not only affect on morphological structure but also affect the genetic structure of eels (Li et al. 2015); (Pavey et al. 2015) and (Laporte et al. 2016).

The research aims at two goals, (1) to examine the genetic characteristics of *A. marmorata* population distributed in Thua Thien Hue, Vietnam and (2) to assess the diversity and building of genetically generated tree for the population of eel populations in the coastal region of Thua Thien Hue for conservation and control of fishing capture. To detect the genetic variability in *A. marmorata*, polymerase chain reaction (PCR) amplification applied and sequencing for the partial 5′ hypervariable region of the cytochrome c oxidase subunit 1 (COI) was carried out as a major and powerful barcoding tool widely applied in animals’ population genetics, phylogenies, and taxonomy (Hajibabaei et al. 2007).

## Materials and methods

### Ethics statement

All animal protocols were approved by the Committee on the Ethics of Animal Experiments of Hue University, Vietnam (permit No. DHH2019-02-113), and were performed strictly with the Guide for fishing capture and animals of Institute of Biotechnology. Animals were fishing capture by fishermen and allowed the Provincial Department of Fisheries.

### Animal preparation and sampling

**Fig. 1:**
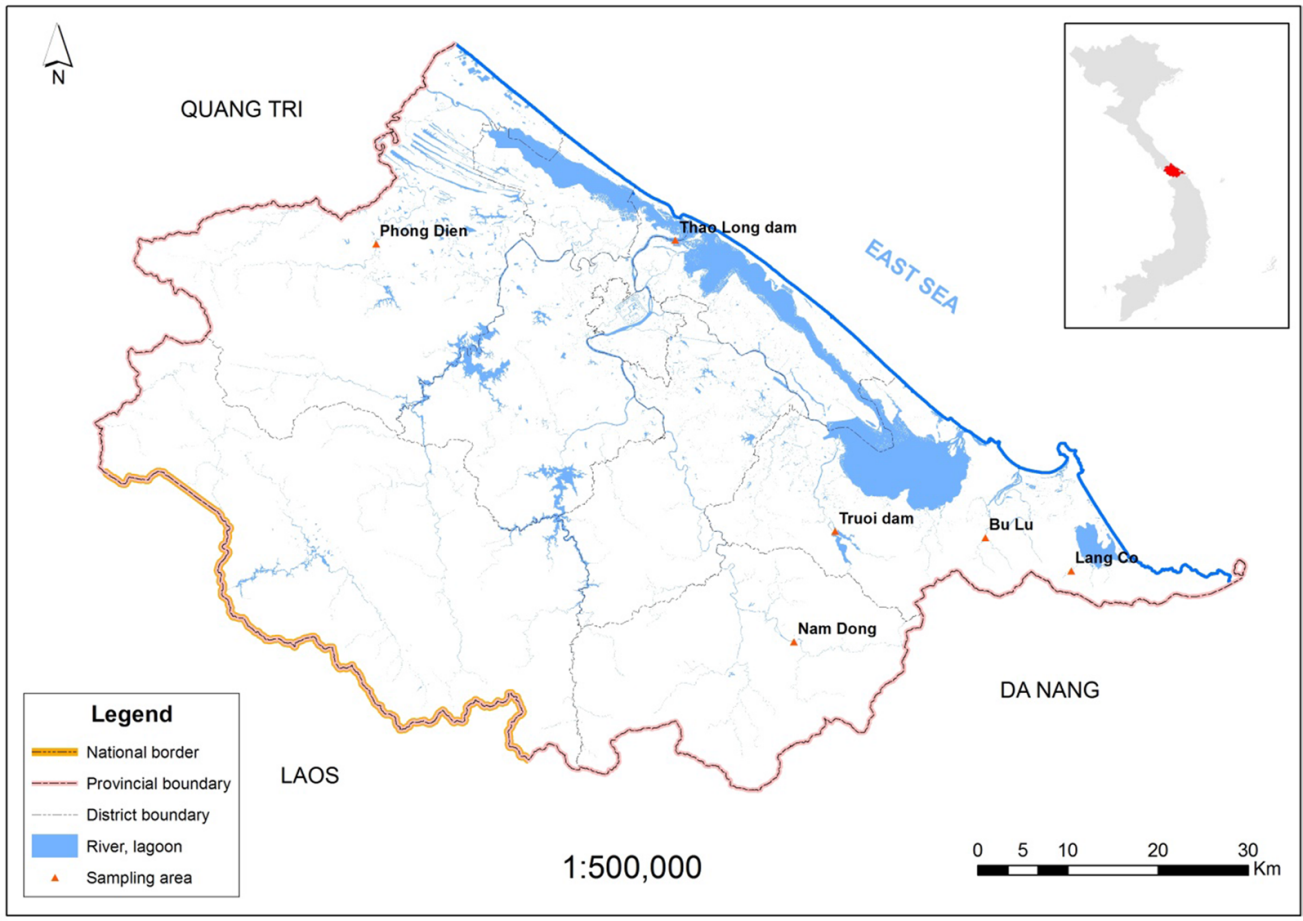
Sample sites of *Anguilla marmorata* in Thua Thien Hue, Viet Nam

Natural adult specimens of *Anguilla marmorata* were collected from 5 localities and indicated in Fig. 1 and number of individuals were conducted at Table 1: Phong Dien (HuePD), Thao Long dam (HueDTL), Truoi dam (HueDTR), Nam Dong (HueND), Bu Lu (HueBL) and Lang Co (HueLC). There were 48 samples were collected from October 2017 to May 2019. Tissues from the adductor muscle were dissected from fresh specimens, preserved in 95% ethanol, and frozen at -80 °C until DNA extraction.

**Table 1:** Location, number, and codes of *Aguilla marmorata* used in the study

No.	Sample collected Location	48 samples	Name of sample	GenBank accession number
1	Thao Long dam	10	HueDTL01, HueDTL02, HueDTL03, HueDTL04, HueDTL05, HueDTL10, HueDTL13, HueDTL21, HueDTL25, HueDTL28	MN067923–MN067932
2	Truoi dam	05	HueDTR01, HueDTR02, HueDTR03, HueDTR04, HueDTR05	MN067933–MN067937
3	Nam Dong	09	HueND01, HueND02, HueND03, HueND04, HueND05, HueND09, HueND14, HueND15, HueND16	MN067938–MN067946
4	Phong Dien	14	HuePD02, HuePD03, HuePD04, HuePD05, HuePD06, HuePD07, HuePD08, HuePD09, HuePD10, HuePD12, HuePD13, HuePD15, HuePD19, HuePD21	MN067947–MN067960
5	Phu Loc (Bu Lu and Lang Co)	10	HueBL01, HueBL02, HueBL07, HueBL08, HueBL15, HueBL18, HueBL20, HueBL22, HueLC01, HueLC02	MN067961–MN067970

### Genomic DNA isolation and amplification

Total genomic DNA was extracted by a description of Kumar et al. (2007) with a modified for *Anguilla marmorata*. The nucleotide sequence of the primers is A.marFw-1: 5′-GCACTAAGCTTCTAATCCG-3′ and A.marRv-1: 5′-GATGATTATTGTGGCAGAAG-3′ (Kumar et al. 2018). The amplification reactions were performed in a total volume of 35 μL. Reaction components: 1 µL DNA, 1 µL F-primer (10 mM), 1 µL R-primer (10 mM), 7 µL PCR buffer (10×), 0.5 µL dNTP (10 mM), 0.2 µL Enzyme Taq (5 UI/µL) and sterile distilled water addition to full volume 35 µL. PCR amplification was performed on the machine (MJ-MiniTM Persanol Thermal Cycle, Bio-Rad) according to the following thermal cycling: 95 °C/5 min; followed by 30 cycles: 95 °C/45 s, 51 °C/30 s, and 72 °C/1 min; The last is 72 °C/7 min. PCR product quality was checked by running electrophoresis on 1% agarose gel with buffers used as TAE 1X (Tris–acetate 40 mM + EDTA 1 mM) and stained with ethidium bromide fluorescent dye (EtBr 0, 5 µg/L), wash the gel with distilled water for 10 min. The gel is observed by the Gel Documentation image analysis system.

### Sequence alignment and molecular phylogenetic analysis

The PCR products of the COI gene region are purified by the Isolate II PCR kit and Gel (Bioline). The PCR product was then sequenced directly by the dideoxy terminator method on ABI PRISM^®^ 3100 Avant Genetic Analyzer (Applied Biosystems). The nucleotide sequences were arranged based on the ClustalW program (Larkin et al. 2007) and the phylogenetic trees showing a genetic relationship built by MEGA. X software (Kumar et al. 2018), based on three methods: Maximum Likelihood (ML), Neighbor-Joining, and Maximum Parsimony with a bootstrap value of 1000 times. The final consensus sequences were submitted to the NCBI (The National Center for Biotechnology Information) database with accession number from MN067923 to MN067970. The phylogenetic study also included COI sequences of 02 other individuals: *A. marmorata*-AP007242.1 and *A. marmorata*-HQ141374.1 taken from GenBank.

## Results

Data were analyzed of 950 bp from 843 bp and conducted the common length of the 48 sequences. The BLAST—Basic Local Alignment Search Tool conducted on NCBI and was used to verify and compare with the sequences of *A. marmorata* (code: HQ141374.1 and AP007242.1), showed that the nucleotide sequences having coverage rates of 100% and 99.76% respectively. There were rates of nucleotides in the COI gene segment of *A. marmorata* is four basic nucleotides equally, except for A (Adenine), which is slightly higher than that of others (Table 2).

**Table 2:** Nucleotide composition of *Anguilla marmorata’*s *COI* gene fragment in Thua Thien Hue, Viet Nam (*A. marmorata* – TTH)

Sample	Quantity (the values in parentheses are calculated in %)				Total (base pairs)	G + C (%)
	T	C	A	G		
*A. marmorata*-HQ141374.1	255 (30.24)	212 (25.14)	229 (27.16)	147 (17.44)	843	359 (42.58)
*A. marmorata*-AP007242.1	254 (30.13)	212 (25.15)	230 (27.28)	147 (17.44)	843	359 (42.59)
*A. marmorata* –TTH	253 (30.03)	212 (25.15)	232 (27.49)	146 (17.43)	843	358 (42.58)

There were no difference in the number of C (Cytosine) nucleotides between *Anguilla marmorata* in Thua Thien Hue compared with two control samples from GenBank (212 correspondings to 25.15%). The number of C and G (Guanine) of eel in Thua Thien Hue is lower than 1 nucleotide compared to two GenBank samples (235 and 146 respectively for C and G). The number and ratio are higher than that of GenBank samples (232 and 27.49%, respectively). The G + C ratio was found lower than A + T (Thymine) in all observed samples (Table 2).

The estimate the maximum substitutability of nucleotides estimated by Tamura and Nei (1993). This resulted in 48 nucleotide sequences with 843 positions in the final dataset and were conducted in MEGA X (Kumar et al. 2018). The results in Table 3 show that the replacement ability of A and G is highest with 22.45%. The substitution between T and G; C and G have the lowest rates of 2.72%.

**Table 3:** Estimate the maximum substitutability of nucleotides according to Gamma parameters (%) of *Anguilla marmorata*’s COI in Thua Thien Hue, Viet Nam

From/To	A	T	C	G
A	–	4.7176	3.9502	14.1647
T	4.3173	–	14.5687	2.7240
C	4.3173	17.3989	–	2.7240
G	22.4495	4.7176	3.9502	–

The deduced amino acid sequences did not exhibit stop codons interrupting the normal amino acid sequence of the real gene. This confirmed the absence of nuclear copies of mitochondria COI gene (NuMTs—Nuclear mitochondrial DNA) (El-Nabi et al. 2017). The amino acids composition percentage in the obtained sequences of the Thua Thien Hue, Vietnam *A. marmorata* partial COI gene fragment were 10.22 for Alanine, 0 for Cysteine, 3.28 for Aspartate, 1.46 for Glutamate, 7.30 for Phenylalanine, 9.85 for Glycine, 2.92 for Histidine, 3.17 for Isoleucine, 1.09 for Lysine, 13.52 for Leucine, 2.15 for Methionine, 3.28 for Asparagine, 6.57 for Proline, 1.82 for Glutamine, 1.46 for Arginine, 5.11 for Serine, 6.20 for Threonine, 7.30 for Valine, 0 for Tryptophan and 3.28 for Tyrosine. The percentage of amino acids in the obtained chains of the COI gene segment of *A. marmorata* in Thua Thien Hue, Vietnam is almost identical with the amino acid composition of samples from GenBank (code: AP007242.1 and HQ141374), except Isoleucine, Leucine, Methionine, Proline and Arginine (Table 4).

**Table 4:** Amino acids composition of COI of *Anguilla marmorata* in Thua Thien Hue, Viet Nam

Amino acids	AP007242.1	HQ141374.1	*A. marmorata*-TTH
Alanine	10.22	10.22	10.22
Aspartate	3.28	3.28	3.28
Glutamate	1.46	1.46	1.46
Phenylalanine	7.30	7.30	7.30
Glycine	9.85	9.85	9.85
Histidine	2.92	2.92	2.92
Isoleucine	13.14	12.77	13.17
Lysine	1.09	1.09	1.09
Leucine	13.50	13.87	13.52
Methionine	2.19	2.19	2.15
Asparagine	3.28	3.28	3.28
Proline	6.57	6.57	6.58
Glutamine	1.82	1.82	1.82
Arginine	1.46	1.46	1.45
Serine	5.11	5.11	5.11
Threonine	6.20	6.20	6.20
Valine	7.30	7.30	7.30
Tyrosine	3.28	3.28	3.28

**Fig. 2:**
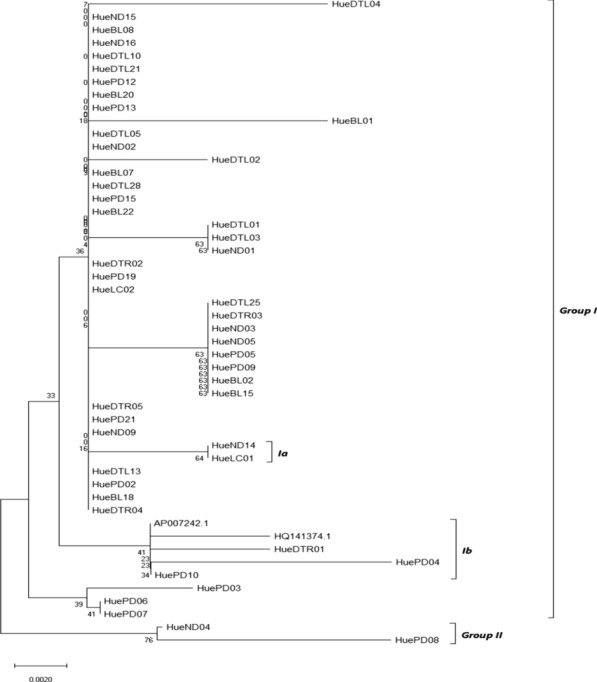
Neighbor-joining phylogenetic tree based on the COI nucleotide sequences

**Fig. 3:**
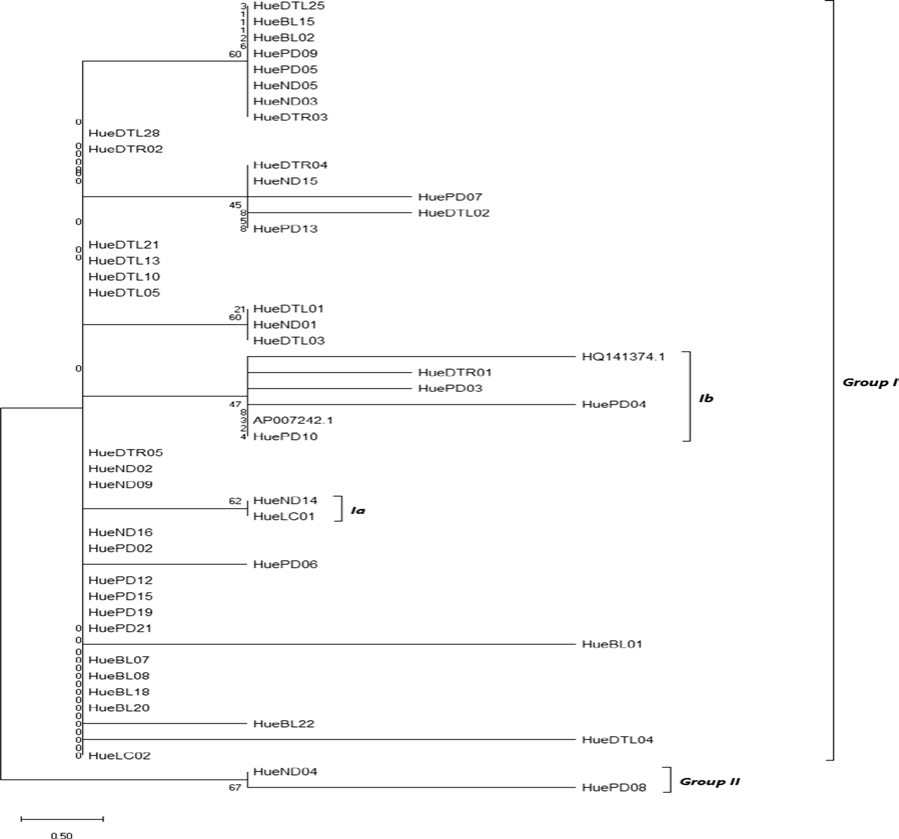
Maximum likelihood phylogenetic tree based on the COI nucleotide sequences using the Tamura-Nei model (Tamura and Nei 1993)

**Fig. 4:**
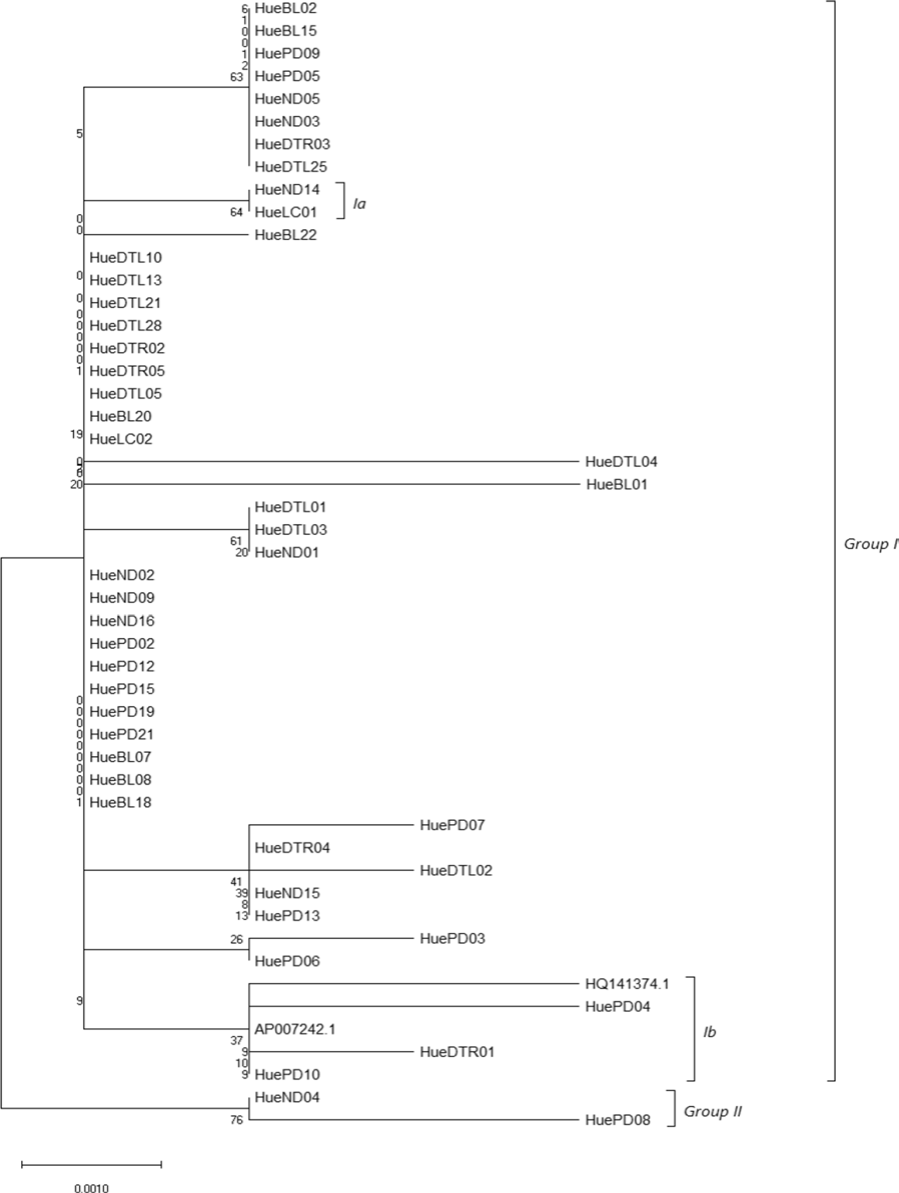
Maximum parsimony phylogenetic tree based on the COI nucleotide sequences

The analysis involved 48 nucleotide sequences of COI of Anguilla marmorata collected in Thua Thien Hue, Vietnam. There was a total of 843 positions in the final dataset. The COI sequence of *A. marmorata*-AP007242.1 and *A. marmorata*-HQ141374, from GenBank. The phylogenetic tree by Neighbor-joining, Maximum Parsimony and Maximum likelihood methods revealed two clad strongly supported with the bootstrap values (1000 replies) on MEGA X software shown in Figs. 1, 2 and 3. The optimal tree with the sum of branch length = 0.08396512 and is shown in Fig. 2. The tree is drawn to scale, with branch lengths in the same units as those of the evolutionary distances used to infer the phylogenetic tree. The evolutionary distances were computed using the Nei-Gojobori method (Nei and Gojobori 1986) and are in the units of the number of synonymous substitutions per synonymous site. For the Maximum Likelihood method, genetically generated trees are based on the Tamura-Nei model (Tamura and Nei 1993). The tree with the highest log likelihood (-1336.57) is shown. The percentage of trees in which the associated taxa clustered together is shown next to the branches. Initial tree(s) for the heuristic search were obtained automatically by applying Neighbor-Join and BioNJ algorithms to a matrix of pairwise distances estimated using the Maximum Composite Likelihood (MCL) approach and then selecting the topology with superior log likelihood value. The tree is drawn to scale, with branch lengths measured in the number of substitutions per site (Fig. 3). The genetic tree was built based on the Maximum Parsimony method (Fig. 4) using the Subtree-Pruning-Regrafting (SPR) algorithm (Nei and Kumar 2000) with a search level 1 in which the initial trees were obtained by the random addition of sequences (10 replicates) with consistency index is 0.727273, the retention index is 0.875000, and the composite index is 0.770000 for all sites and parsimony-informative sites (in parentheses). The percentage of replicate trees in which the associated taxa clustered together in the bootstrap test (1000 replicates) are shown next to the branches (Felsenstein 1985). The proportional tree, expressed with branch length, is calculated using the moving average method and measured in the number of substitutions per site. Estimates of the evolutionary divergence between the COI nucleotide sequences were made among 50 sequences indicate that all samples of *A. marmorata* collected in Thua Thien Hue have farther genetic gaps with the two eel samples from gene bank data (0.005–0.012) than those of themself. Except for HueND15, there is an exceptionally low genetic distance of 0.000 to *A. marmorata*-AP007242.1.

The results from Figs. 2, 3, and 4 also indicated that all specimens have a significant relation and separated into 2 groups. Group I with 48 individuals, 46 from Thua Thien Hue and 2 form GenBank (code: AP007242.1 and HQ141374). AP007242.1, HQ141374, and some samples from Thua Thien Hue (HueDTL01, HuePD04, and HuePD10), expect in Maximum Parsimony tree include HuePD03, sophomore clustered together as sister group and shows their close relationship. Group Ib, individuals grouped into separate clusters and have a relatively long genetic distance from the group of other individuals. Group II consists of two individuals ND04 and PD08 with relatively large genetic ranges in the Neighbor-Joining and Maximum Likelihood models (Figs. 2 and  3), while in the trees producing Maximum Parsimony, group II is replaced by two individuals belonging to group Ia of the other two models are HueND14 and HueLC01 with equal genetic distance. In the Maximum Parsimony model, HueND04 and HuePD08 were replaced by group Ia positions of HueND14 and HueLC01. Although there are differences in the location of individuals on genetically generated plants, there is uniformity in the genetic distance between them in the general population. Although there are differences in the location of individuals on genetically generated plants, there is uniformity in the genetic distance between them in the general population.

In addition, among individuals in the eel population in Thua Thien Hue, there is a certain difference in origin compared to individuals from the gene bank. This may be related to the species migration process. After the parent’s spawn, the young eels will follow the currents to the estuaries where their parents used to live (Arai 2016) have formed the characteristic specific to the population *A. marmorata* in Thua Thien Hue. This shows that the ability to preserve the species characteristics during the migration of the *Anguilla marmorata* eel from the ocean to the continent is remarkably high. This study is of great importance in establishing strategies for conserving and developing the gene for eel in Vietnam and Thua Thien Hue.

## Discussion

Using DNA barcode to identify and genetic diversity of species is being researched and contributed significantly by scientists around the world. The short DNA sequencing used as a barcode for animals promises to provide an accurate, efficient species identification tool and identifying species with intact specimens, young specimens difficult to identify species by morphology (Kress et al. 2015).

Tautz et al. (2003) created the case for a DNA-based classification system (Tautz et al. 2003). Hebert et al. (2003) proposed that a single gene sequence would be sufficient to distinguish all, or at least the vast majority of animals, and proposed the use of mitochondrial I (cox1) DNA cytochrome oxidase subunits such as a global biological identification system for animals. Sequences are likened to a barcode, with species identified by a specific sequence or by a tight cluster of terribly similar sequences (Hebert et al. 2003), (Hebert and Barrett 2005). Once the global COI barcode database has been established for fish, this will be an invaluable tool for fisheries managers, will be an invaluable tool for fisheries managers, aquatic ecologists, and fish retailers, and for those who want to distribute develop micro fish identification. The scientific and practical benefits of fish barcodes are truly diverse (Ward et al. 2005). The results of our study analyzed the characteristics of a COI gene segment on eel flower isolated in Thua Thien Hue, Vietnam. This gene segment can be used as a genetic barcode to identify and evaluate genetic diversity for eels in Vietnam and the world.

Diversity and a close relationship with two individuals from GenBank were found in three different phylogenetic trees of the giant mottled eel *Anguilla marmorata* in Thua Thien Hue, Viet Nam can be attributed to several issues, most of which are based on life-history events of the species. First, during reproduction of adults and at the onset of larval migration, *A. marmorata* populations gain effective chances for mixing. Until now, spawning areas of *A. marmorata* suggested that occurred in the same oceanic areas as other anguillids, such as *Anguilla japonica* (Pous et al. 2010), (Tsukamoto et al. 2011). The spawning areas have not been well identified yet (Kuroki et al. 2008), (Pous et al. 2010), (Réveillac et al. 2008), (Robinet et al. 2008), except for the North Pacific population. So, all its collected samples have the same origin and belong to the same genetic pool. Second, being the catadromous migration and the long migration loop noticed in *A. marmorata* life (Arai 2016) could increase the probability of mixing of larvae during the migration from the Sargasso Sea to continental drift (Pujolar et al. 2009). It seems that the same process applies also to Thua Thien Hue, Viet Nam populations due to the close identity of all specimens in Thua Thien Hue with their counterparts in Taiwan and the Pacific Ocean (taken from GenBank). Third, before going up the rivers *A. marmorata* glass eels stay offshore for 3 months before moving into estuaries which might increase the mixing of individuals (Pujolar et al. 2009).

However, some determinant variables appear in their life and preclude the attaining of a real state “panmixia” of the populations (El-Nabi et al. 2017). Of these variables, spawning cohorts exhibit large variations in time of maturity, the latitude where the population exists and the characteristics of the population there, the onset of migration, and the arrival time to the Sargasso Sea. The yellow eel growth stage may be as short as two to three years in warm productive habitats, but about six to 20 years or more in more in different locations (Williamson and Boëtius 1993). *A. marmorata*, a catadromous eel, migrates upstream on nights, following the lunar cycle (Wang et al. 2014). The dramatic environmental changes between ocean and freshwater during particular phases of their life cycle shape their physiological features, e.g. visual sensitivity, olfactory ability, and salinity tolerance (Wang et al. 2014) all of which can manipulate the onset of the genetic signal. The time of arrival of spawners to the Sargasso Sea also seems to be variable. As a result, spawners from geographically separated areas could differ in the arrival time at the spawning sea areas. This isolating by the time of spawning groups that causes a restriction in gene flow, taking place between early and late spawners (Hendry and Day 2005). This trend leads to differences in the genetic distance of some individuals, such as HueDTL04, HueBL01, HueDTL02, HueND04, HuePD08, with the others.

The results of *A. marmorata* genetics research based on the COI gene segment showed that the *Anguilla marmorata* population in Thua Thien Hue, Vietnam can be divided into two groups indicated the genetic potentials of the species in the vast geographical range in which it lives. In the population structure of *Anguilla marmorata*, Watanabe et al. (2005) suggested at least four subpopulations (North Pacific, Micronesia, Indian Ocean, and South Pacific) with metapopulation structure evident in the Indian Ocean, and South Pacific. Ishikawa et al. (2004) found the differences among geographic samples that revealed the existence of five geographic subpopulations around North Pacific, Madagascar, Sumatra, Fiji, and Tahiti (Ishikawa et al. 2004). Minegishi et al. (2008) were more consistent with the molecular analyses proposing four genetically different subpopulations (North Pacific, South Pacific, Indian Ocean, Guam region), offering that the North Pacific population is fully panmictic with some meta-population structure in the South Pacific and the Indian Ocean populations (Minegishi et al. 2008).

Recently, Gagnaire et al. (2009) showed the existence of three genetically distinct, reproductive *Anguilla marmorata* populations in the North Pacific, South Pacific and Southwest India, showing partial gene isolation occurs due to reproduction, but for some inter-population gene lines it can occur during long-term migration of the species (Gagnaire et al. 2009). Genetic analysis of Donovan et al. (2012) on *Anguilla marmorata* eel on populations distributed in the Pacific Ocean recognition of two lineages distinctive for the eastern Caroline Islands and Guam, and the likelihood of an additional spawning area in the Indo-Pacific Ocean (Donovan et al. 2012). In the first study of phylogenetic relationships and genetic diversity from all *Anguilla* taxa inhabiting Indonesian waters, based on 1115 specimens belong to four *Anguilla* species. The results showed that *A. marmorata* was also split into two clades, supported by a high bootstrap value (Fahmi et al. 2015). This is necessary data for local management and conservation of this valuable resource in terms of both biodiversity and economic development (Fahmi et al. 2015).

## Data Availability

The datasets of COI sequences analyzed during the current study are available on the GenBank with accession number from MN067923 to MN067970. The data were simultaneously made available to ENA in Europe and the DNA Data Bank of Japan.
